# Monomeric CRP is prevalent in the blood of patients with rheumatoid arthritis and correlates with clinically relevant markers

**DOI:** 10.3389/fimmu.2026.1925147

**Published:** 2026-09-18

**Authors:** Alexia Correia Ferreira, Lucia Martin-Gutierrez, Christophe Lalaurie, Karen Feehan, Priyanka Lakhani, Imogen Buckle, George A. Robinson, Elizabeth C. Jury, Jessica J. Manson, Thomas C. R. McDonnell

**Affiliations:** 1 Department of Ageing, Rheumatology and Regenerative Medicine, Division of Medicine, University College London, London, United Kingdom; 2 Department of Rheumatology, University College London Hospital NHS Trust, London, United Kingdom; 3 Haemostasis Research Unit, Institute for Cardiovascular Science, University College London, London, United Kingdom

**Keywords:** C-reactive protein, dissociation, inflammation, rheumatoid arthritis, simulation

## Abstract

**Introduction:**

Elevated C-reactive protein (CRP) is a hallmark of rheumatoid arthritis (RA) flares. CRP exists as pentameric (pCRP) and monomeric (mCRP) isoforms with distinct functions, with pCRP generally pro-inflammatory and mCRP more immunomodulatory. Standard clinical assays do not distinguish between these forms, and it remains unclear whether elevated CRP in RA reflects structural variation. We investigated CRP isoform distribution and function in RA using biomolecular and biophysical approaches.

**Methods:**

Patients with RA (n=39) were recruited from the University College London Hospital. Serum CRP was requantified by commercial ELISA. Western blotting using ELISA antibodies determined detectable CRP isoforms. mCRP was assessed using a specific monoclonal antibody by ELISA and western blot. Molecular dynamics simulations of CRP were conducted at 30 °C, 37 °C, and 50 °C to evaluate structural stability and dissociation. CRP stability was further assessed under NaCl and CaCl_2_ gradients. Functional effects were evaluated by spectral flow cytometry.

**Results:**

Commercial ELISA correlated with clinical CRP measurements (p=0.002, r=0.39) but did not detect mCRP by ELISA or by Western blot. Presence of mCRP was confirmed in RA serum using an mCRP-specific antibody by ELISA and Western blot and was significantly elevated compared to controls. mCRP levels correlated with anti-CCP antibodies (p=0.02, r=0.4). pCRP dissociation was unaffected by ionic gradients. Simulations showed reduced pCRP stability at 37 °C compared to 30 °C and 50 °C, suggesting increased structural fluctuation. Higher mCRP levels were associated with suppression of CD11c expression by flow cytometry.

**Discussion:**

Standard assays fail to detect mCRP in RA. Elevated mCRP and its associations with autoimmunity and cellular markers suggest it may contribute to disease mechanisms and represent a clinically relevant biomarker.

## Graphical abstract

**Figure:**
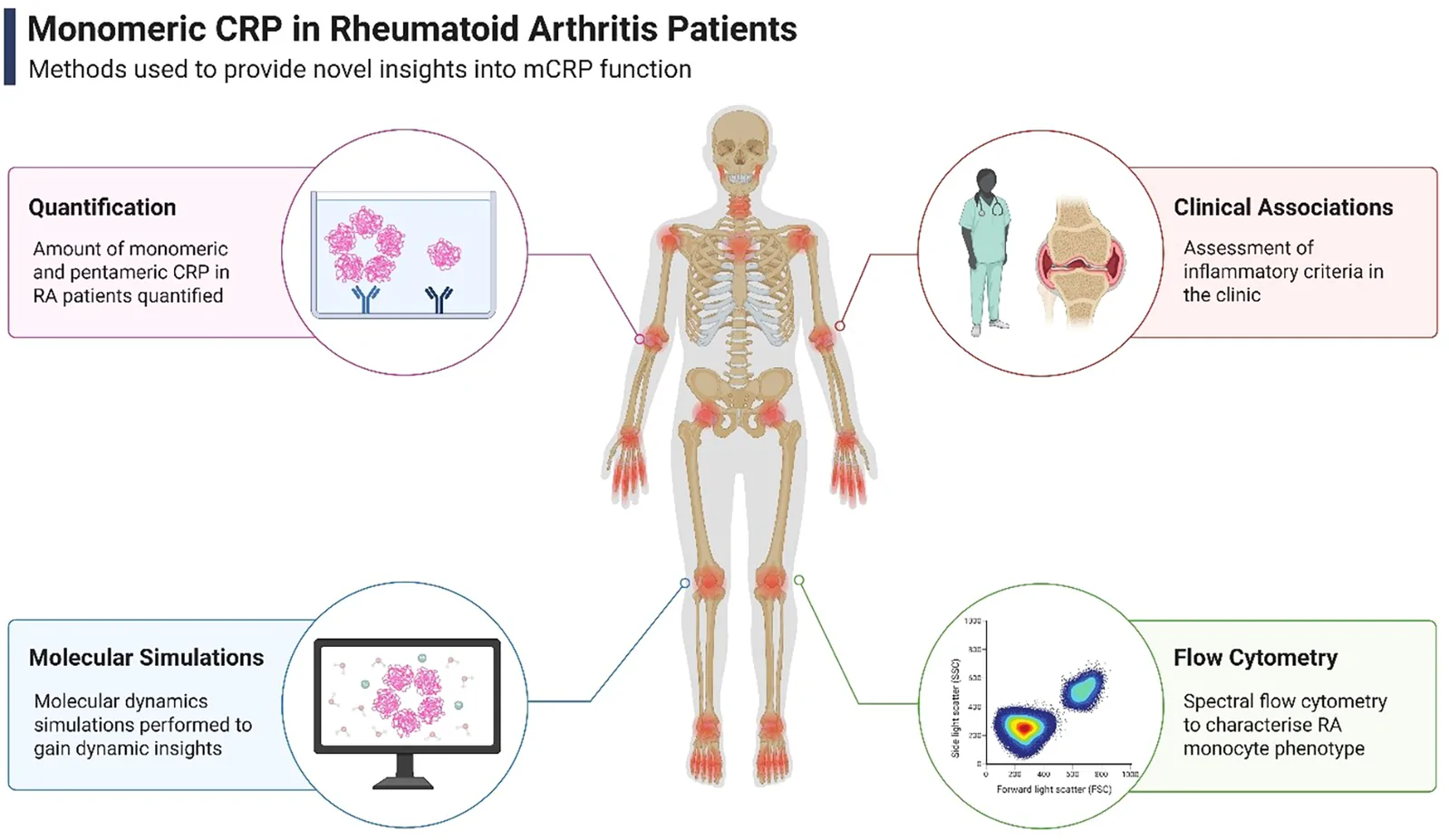
This project combines molecular immunological techniques with molecular simulation data and patient data to fully characterise the levels and significance of mCRP in people with RA.

## Introduction

Rheumatoid arthritis (RA) has a global prevalence of ~1% (1) and is characterised by autoimmune-driven chronic inflammation of the synovial joints, leading to cumulative joint damage and a risk of systemic complications. Acute-phase proteins such as C reactive protein (CRP) are used to help define disease activity in indices such as Disease Activity Score 28 (DAS28)—a widely used clinical tool for measuring disease activity in RA (2). CRP is primarily produced and secreted by hepatocytes in response to IL-6, IL-17, and stress signals from vascular tissue and indirectly in response to IL-1b through NF-kB-mediated IL-6 production (3); consequently, patients typically present with elevated CRP levels during disease flares.

Interestingly, CRP exists primarily in two structural forms, one pentameric (pCRP) and one monomeric (mCRP). Far more is known about the role of pCRP in health and disease, likely due to the poor solubility and resulting poor detectability of mCRP in circulation (4). More recent studies comparing these different multimeric states have shown distinct functions, prompting a re-evaluation of their respective contributions to disease pathology and challenging earlier assumptions of CRP’s role in RA (5). A number of these comparisons generate data highly relevant to RA pathogenesis; for example, pCRP has been shown to bind to inflammatory minimally modified low-density lipoprotein, serving to dampen monocyte activation which are crucial to RA disease progression, a function which is notably not observed for mCRP. Additionally, some studies even suggest that in early or preclinical RA, pCRP may even exert disease-protective effects through immune complex formation and complement modulation, a function mCRP would be incapable of due to its structural differences. Furthermore, pCRP has also been shown to drive osteoclast differentiation through RANKL production, contributing to bone erosion. Meanwhile, mCRP may have the opposing effect having been reported to inhibit RANKL-driven osteoclast maturation, potentially reducing bone erosion (5, 6). Overall, this suggests that the balance between mCRP and pCRP may be crucial in modulating erosive disease processes.

Despite this overtly anti-inflammatory function, mCRP can be considered in some way pleiotropic as significant data also highlight the inflammatory potential of mCRP. To this end, mCRP has been shown to increase IL-8 production by neutrophils and support monocyte-dependent T-cell activation, promoting persistent synovial inflammation. Additionally, in RA synovium, mCRP has been shown to activate fibroblast-like synoviocytes through CD32/CD64, triggering NF-kB and p38 MAPK signalling and amplifying inflammation through cytokine release (7). Osteoclasts in RA can also be affected by mCRP, through RANKL inhibition leading to altered osteoclast activation (8). Another signalling pathway mCRP activates is through CD11c, which has several functions in RA. CD11c has a role in the consumption of CD47− cells by CD11c+ dendritic cells (a function believed to be impaired in RA), whereas it can also push monocyte polarisation to a more pro-inflammatory phenotype.

The functional differences of CRP isoforms can be intrinsically linked to the result of dissociation reshaping the protein’s binding interfaces and subsequent activity. pCRP consists of five identical, non-covalently linked subunits in a cyclical arrangement forming a central, hydrophobic pore (9), enabling the formation of two distinct recognition sites, namely, the effector face, binding to complement component 1q (C1q) and a range of Fc gamma receptors, and the binding face mediating calcium-dependent interactions with phosphocholine (PC), demonstrated in **Figure 1**.

**Figure 1:**
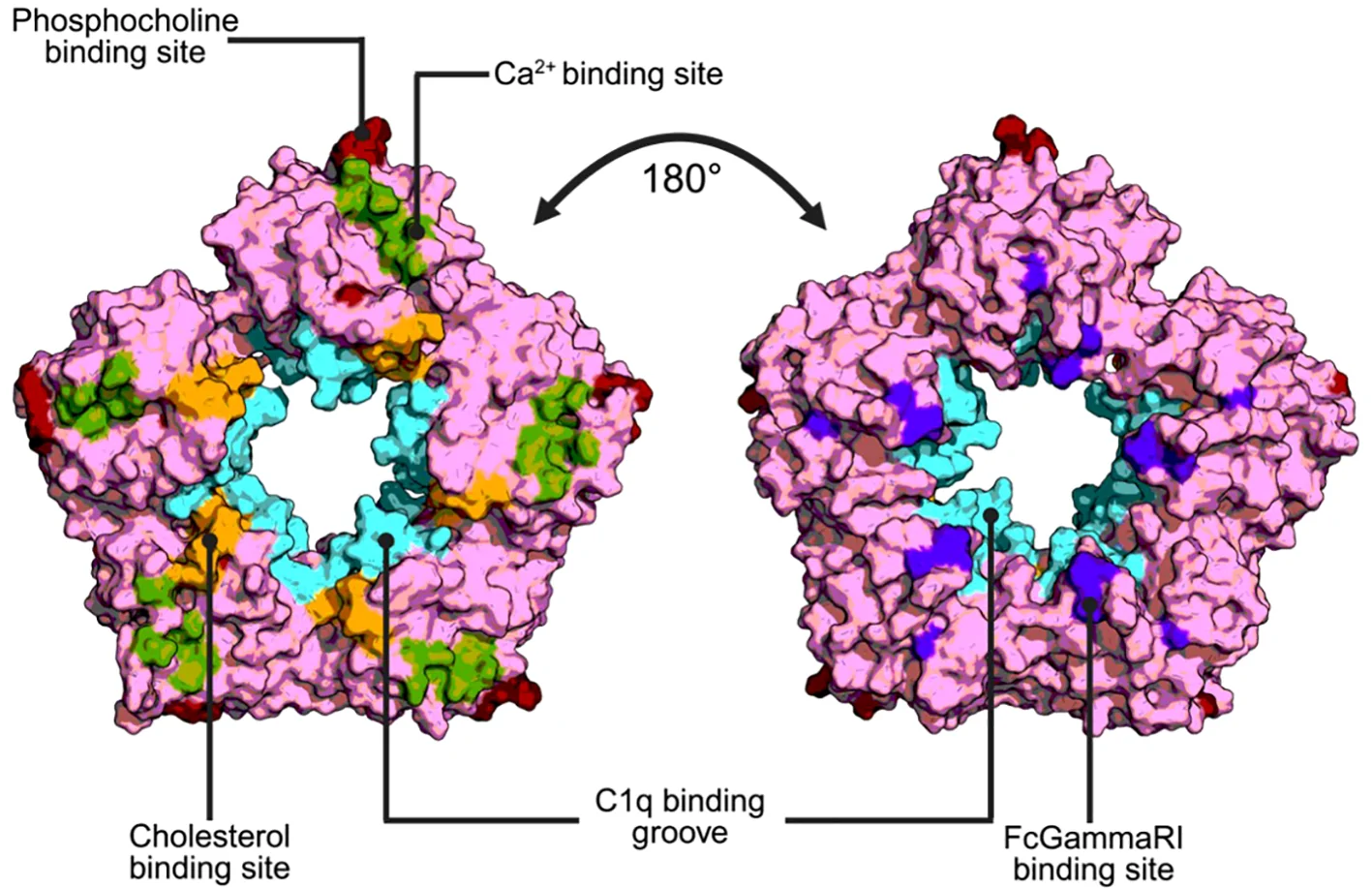
Representation of the crystal structure of CRP (PDB: 1GNH), including binding sites of interest such as c1q (cyan) FcGamma binding (purple), cholesterol binding (yellow) and phosphocholine (burgundy).

The route of production of mCRP is controversial with mCRP canonically arising from pCRP dissociation, often due to pCRP binding to PC residues in lysophosphatidylcholine, a lipid generated by phospholipase A2 on damaged cell membranes (3). However, despite this well-characterised model, dissociation has been difficult to achieve in the laboratory, making studying mCRP difficult. Whilst the monomeric CRP contains the same binding sites as the pentamer, the surface exposure of the C1q binding groove, cholesterol, and FcGammaRIIa binding sites are significantly altered after dissociation. These changes in binding site exposure and function evidence that pCRP and mCRP are not functionally equivalent and raises the necessity of isoform-specific characterisation in RA to fully characterise pathogenesis.

We present a thorough characterisation of mCRP and pCRP in a cohort of people with RA, with the aim to elucidate the significance of mCRP on underlying RA pathogenesis. We aim to achieve this by identifying if CRP assays are blind to monomeric CRP, and if mCRP has links to clinical parameters, and then identify both the potential cellular signal of mCRP and the potential pattern of dissociation seen by molecular dynamics of CRP’s unfolding.

## Methods

### Cohort

Patients with seropositive RA (n=39) who fulfilled the 2010 EULAR/ACR classification criteria for rheumatoid arthritis were recruited from the University College London Hospital NHS Trust rheumatologist-led ultrasound (US) clinic. Patients had synovitis confirmed on US, with power Doppler confirmed synovitis present in at least one joint. Exclusion criteria included the following: presence of comorbidities such as cancer, infection, vaccination 3 months prior to enrolment, pregnancy or lactation, and use of anti-IL-6 therapies. Healthy adults were also recruited as healthy controls (HC) (n=11). Peripheral blood collected from both patients and HCs was processed for serum as per the International Society on Thrombosis and Haemostasis (ISTH) guidelines and for PBMC isolation using SepMate tubes as per the manufacturer’s instructions. Baseline serology/immunology variables at the time of recruitment were collected and stored in line with General Data Protection Regulations GDPR. Participant demographic and clinical features are displayed in **Table 1**.

**Table 1:** Patient demographics for RA patients and healthy controls.

	RA	HC	P value
Number (n)	39	11	NA
Age, years: mean, SD (range)	53.17, 17.28 (25-84)	38.25, 7.62 (26-49)	0.0216
Sex (F:M)	(30:9)	(7:4)	0.1909
Disease duration (years): median, IQR (range)	6, 15 (1-43)	NA	NA
CRP, NR >5 mg/L: median; IQR (range)	3; 9.975 (0.6-80.9)	NA	NA
Rheumatoid factor, IU/mL: mean, SD (range)	83.73, 255.52 (10-1209.9)	NA	NA
CCP antibodies units/mL: median; IQR (range)	165.5; 306.25 (0.9–463)	NA	NA
Erythrocyte sedimentation rate, NR 0–20 mm/hr: median, IQR (range)	10, 23 (2–134)	NA	NA
White cell count, NR 3.0-10.0 × 10^9^ g/L: median, IQR (range)	6.93, 3.01 (3.91–11.68)	NA	NA
Neutrophils, NR 2.0-7.5 × 10^9/^L: median, IQR (range)	4.67, 2.4 (1.32–9.19)	NA	NA

IQR, interquartile range; NR, normal range.

### Structural separation

5 µL of serum was added to 100 µL 1× PBS and transferred to 0.5-mL centrifugal filter units (Merck cat: UFC505096, NWMCO 50 kDa) in 2-mL Eppendorf tubes. Tubes were centrifuged at 10,000×g for 30 min, and the fraction below the membrane was kept as monomeric CRP. The fraction above the membrane was then transferred to a new 2-mL Eppendorf tube, and 100 µL 1× PBS was added to the centrifugal column. Samples were re-spun, added to 100 µL of 1× PBS, and kept as non-monomeric CRP.

### ELISA

The commercial ELISA was performed as per the manufacturer’s instruction (DY1707; Bio-Techne). Briefly, 100 µL of detection Ab was immobilised onto a 96-well strip plate and incubated overnight at 4°C. This was then followed by 2-h incubations at room temperature with 300 µL of 1% BSA, 100 µL of samples (whole serum or fractionated CRP serum in 1:12,000 or 1:600 dilution in 1% BSA, respectively), 100 µL of capture Ab, and finally 100 µL of streptavidin HRP (1:200 in 1% BSA). Finally, 100 µL of substrate solution (KPL) was added, and colour allowed to develop for 10 min before quenching with 50 µL of stop solution. After each incubation step, wells were washed three times with 300 µL of 0.1% PBS-Tween20 (PBS-T). Absorbance at 450 nm was then measured using a Tecan microplate reader, and standard curve-based quantification of samples was used to determine the CRP concentration.

For monomeric quantification, we utilised a published methodology (10); 100 µL mouse anti-human mCRP mAb CRP-8 (Sigma) was immobilised onto a MaxiSorp 96-well plate overnight at 4°C. Wells were blocked with 300 µL of 5% BSA-PBS at 37°C for 2 h, followed by 1-h incubations at room temperature of 100 µL of sample (1:10 serum dilution in 5% BSA-PBS), 100 µL of sheep anti-human CRP polyclonal antibody (CTA-1099 (Creative Labs); 1:2,000 in 5% BSA-PBS), and 100 µL anti-sheep IgG horseradish peroxidase-conjugated Ab (HAF016; 1:10,000 in 5% BSA-PBS). Finally, 100 µL of substrate solution was added, and the reaction was left to proceed for 20 min before quenching with 50 µL of stop solution. After each incubation step, wells were washed three times with 300 µL of 0.01% PBS-T. Absorbance at 450 nm was then measured using a microplate reader.

### Molecular simulations

C-reactive protein was modelled using the crystal structure PDB ID: 1GNH through the CHARMM-GUI Glycan Reader and Modeler website (https://www.charmm-gui.org/). Simulations were carried out at 303.15, 310.15, and 323.15 K (30°C/37°C/50°C, respectively) with an initial equilibration stage in constant temperature, particle number, and volume using the CHARMM36 force field with NAMD (11, 12). Simulations were carried out as previously (13, 14). Briefly, the TIP3P model was used for explicit water molecules with a cubic system size of at least 10 Å from the protein in each axis with 0.15 M salt. All calculations were performed at 303.15 K before running. The particle mesh Ewald algorithm was applied to calculate electrostatic forces, and the van der Waals interactions were smoothly switched off at 10 Å by a force-switching function (15). To assure gradual equilibration of the system, positional restraints for backbone and side-chain heavy atoms were applied and the restraint forces were gradually reduced during the equilibration. For the production NPT simulation, the Langevin coupling coefficient was set to 1 ps^−1^ and a Nosé–Hoover Langevin piston was used to maintain constant pressure (1 bar) with a piston period of 50 fs and a piston decay of 25 fs. The time step was 2 fs, coordinates were saved every 100 ps, and each simulation was repeated five times up to 150 ns per replicate. Trajectory analyses—including root mean square deviation (RMSD), dynamic cross-correlation matrix (DCCM), radius of gyration (R_G_), and principal component analysis (PCA)—were conducted using R scripts both independently and collectively for temperature conditions; visualisations were carried out in PyMOL and VMD.

### Western blotting

Samples (20 µL + 5 µL LDS dye) were loaded in 4%-12% SDS-PAGE gels (Bis–Tris) and run for 32 min at 165 V. Proteins were transferred to PVDF membranes using the Trans-Blot Turbo Transfer system at 25 V for 7 min (Bio-Rad). Membranes were blocked in 5% milk for 1 h at room temperature. Membranes for the dissociation experiment were cut above the 38-kDa marker; all others remained intact throughout blotting. Both membranes were probed overnight with relevant Ab in 1:1,000 dilution in 5% milk-PBS, >38 kDa with the human CRP DuoSet ELISA detection antibody (Bio-Techne cat: DY1707), whereas <38 kDa with anti-human mCRP mAb (CRP-8). The membranes were washed (PBST 0.1%), and signal was detected with anti-mouse-HRP (Dako, 1:1,000). Exposure was done using ECL (Amersham) and an ImageQuant 800 chemiluminescent system.

### Coomassie staining

Samples were loaded as per Western blotting above and then stained with InstantBlue Coomassie protein stain (Abcam) overnight. Colorimetric imaging was then performed using the Amersham ImageQuant 800 chemiluminescent dock.

### Flow cytometry

Fresh PBMCs were isolated from patients with RA using Ficoll-based density centrifugation performed using SepMate tubes (STEMCELL) before storage in LN. Cells were thawed for staining, and for exclusion of dead cells from analysis, cells were incubated in the dark for 30 min with a dilution of 1:1,000 with Zombie NIR™ (BioLegend) at 4°C. Fc receptors were blocked using Human TruStain FcX FC receptor blocking solution (BioLegend; 1:100) for 30 min at 4°C to prevent non-specific binding of fluorescent antibodies. The following antibodies against the indicated antigen were used at 1:100 dilution to identify immune cell populations, monocyte phenotype, and cytokine secretion: CD45-SparkViolet538 (HI30;BioLegend), CD11b-SparkUV387 (ICRF44; BioLegend), CD3-BUV395 (UCHD1; BD Biosciences), CD56-StarBrightUV575 (QA17A16; BioLegend), HLA-DR-BUV805 (G46-6; BD Biosciences), CD14-BV421 (M5E2; BioLegend), CD16-APC-Fire-810 (S18012E; BioLegend), CD11c-BV711 (B-Ly6; BD Biosciences), CD163-StarBrightUV445 (EDHu-1; Bio-Rad), CCR2-BUV496 (LS132.1D9; BD Biosciences), CD142-BUV615 (HTF-1; BD Biosciences), CD55-BUV737 (IA10; BD Biosciences), CD206-BV480 (19.2; BD Biosciences), CD68-BV785 (Y1/82A; BioLegend), CD80-Real Blue 545 (B7-1; BD Biosciences), CD97-Real Yellow 610 (VIM3b; BD Biosciences), CX3CR1-Real Yellow 703 (2A9-1; BD Biosciences), CD86-Pe/Fire810 (IT2.2; BioLegend), MerTK-APC (590H11G1E3; BioLegend), CD36-AF700 (5-271; BioLegend), CD64-APC/Fire810 (S18012E; BioLegend), IFNγ-BV570 (4S.B3; BioLegend), TNFα-BV605 (Mab11; BioLegend), IL-1β-BV650 (CRM56; eBioscience), IL-4-Real Blue 780 (MP4-25D2; BD Biosciences), IFNα-PE-Cy5 (7N4-1; BD Biosciences), and IFNβ-PE/Cy5 (A1; Thermo Fisher) conjugated with the Abcam PE/Cy5 conjugation kit (Lightning Link^®^ ab102893). Intracellular stains were performed using the Cytofix/Cytoperm™ Fixation and Permeabilization Solution (BD Biosciences) and Permeabilization Buffer (Thermo Fisher Scientific) as per the manufacturer’s guidelines. Spectral flow cytometry data was acquired using the Sony ID7000™ Spectral Cell Analyzer.

### Dissociation

Salt-gradient dissociations utilised a diluted 2-M NaCl stock solution in PBS; dissociations began at 1 M and were diluted through 12 equidistant points to 0.15 M. 2 µL serum was incubated with 60 µL of buffer and incubated either at 4 °C or 37 °C for 80 min. Samples were then analysed through Western blotting or Coomassie staining for CRP structures. Calcium dissociations were carried out identically, starting from a stock of 1 M CaCl_2_ and dilution from 5.5 to 0.3 mM across 12 equidistant points.

### Statistical analysis

Statistical testing was performed using GraphPad Prism software where a Shapiro–Wilk test for normality (alpha=0.05) was conducted on all datasets prior to statistical analysis. In normally distributed data, a one-way ANOVA test was used to determine significance. Conversely, the Kruskal–Wallis test (multiple groups) or Wilcoxon test (two groups) were used. Statistical significance was set at p<0.05, and any statistically significant results have been shown within figures.

Flow cytometry data was analysed in FlowJo™ (v10.8.1), and then statistical significance was then determined using GraphPad Prism 8 software. For comparison between two groups, a two-sided unpaired Students t test was used for parametric data, or a Mann–Whitney test for non-parametric data.

## Results

### Clinical characteristics

Patient characteristics are presented in **Table 1**, and CRP presented is from clinical laboratory. Healthy controls were significantly younger than patients.

Patients were on a range of medications with 7 patients on naproxen (3/11 high mCRP, 4/27 low mCRP), 11 on methotrexate (3/11 high mCRP, 8/27 low mCRP), 5 on sulfasalazine (1/11 high mCRP, 4/27 low mCRP), 11 on HCQ (3/11 high mCRP, 9/27 low mCRP), 8 on Dep-Medrone (4/11 high mCRP, 4/27 low mCRP), 3 on etanercept (all mCRP), and 3 on lefunomide (1/11 high mCRP, 2/27 low mCRP). There was no significant difference between medications between the monomer high and monomer low patients for any of these medications. None of these would directly affect monomerisation of CRP. The only therapy in literature to do this is prednisone which only four patients were on at the time of sampling (3/27 low mCRP vs 1/11 high mCRP). Whilst some of these can effect levels of CRP, none have been shown to effect the distribution of the monomeric vs pentameric CRP.

### Clinical assays do not detect monomeric CRP

Initially, we confirmed that our commercial ELISA (Bio-Techne) mirrored those in the clinical lab with raised CRP in the RA group as expected (**Figure 2a**). Identification of potential structural selectivity of the ELISA was investigated by Western blotting using the ELISA antibodies as detection mechanisms. Both the CRP ELISA capture and detection antibodies recognised predominantly the multimeric forms of CRP (~76 kDa, ~102 kDa) (**Figure 2b**). Further blotting with a monomeric CRP-specific mAb (CRP-8) identified predominantly the monomeric form (<28–31 kDa) (**Figure 2C**) with a small recognition of a potentially larger, modified form ~38 kDa. To confirm the presence of monomeric CRP in these samples, a monomeric CRP-specific ELISA was performed, confirming higher levels of mCRP as well as pCRP in patients with RA compared with healthy controls (**Figure 2d**).

**Figure 2:**
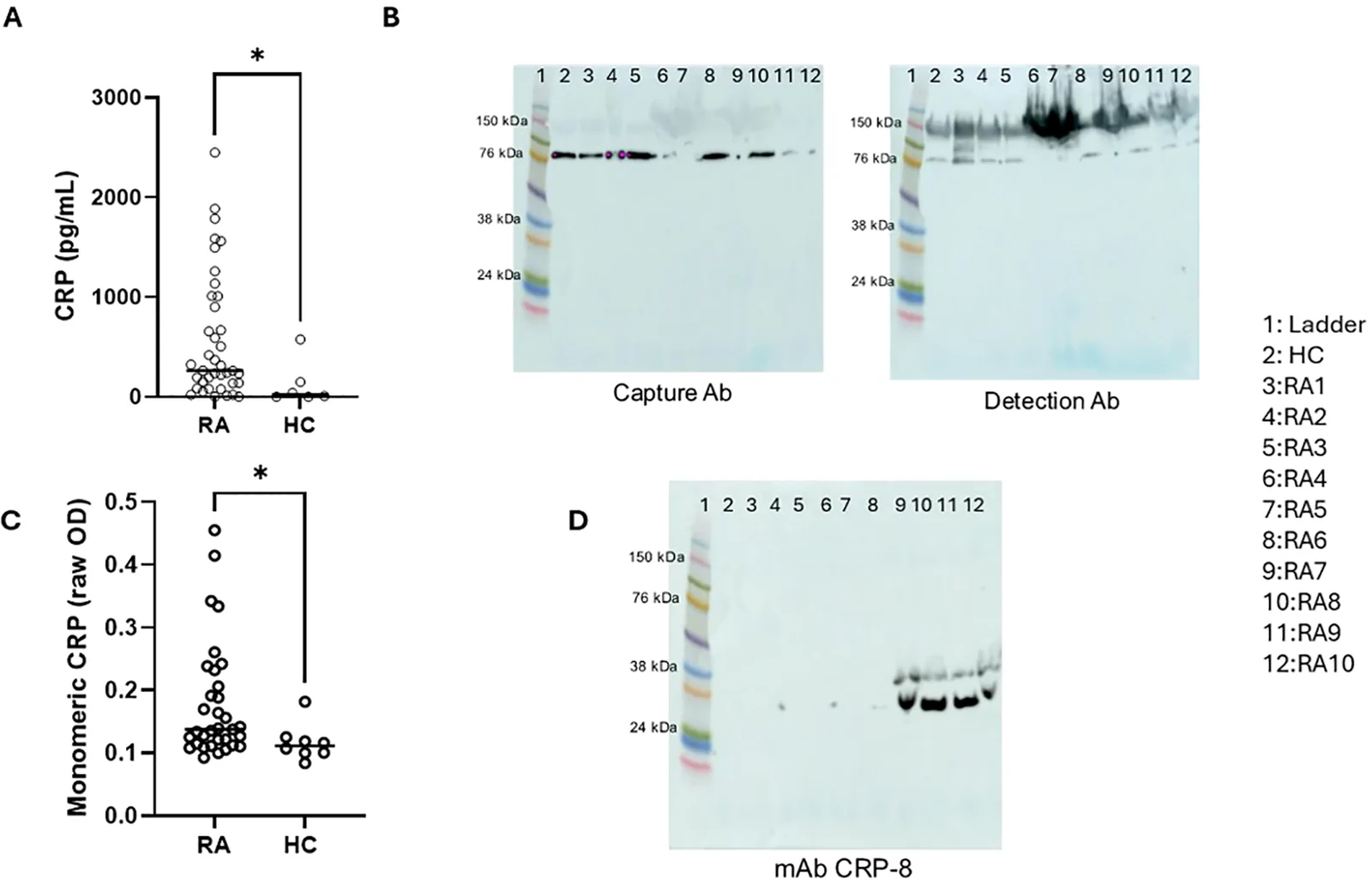
Identification of structural specificity for clinical assays. To assess potential structural specificity within clinical assays, we utilised a clinical sandwich assay (Bio-Techne) for CRP detection **(A)**. This study aims to identify patients (n=45) into groups matching clinically defined high CRP and low CRP groups and healthy control **(A)**; and to identify potential structural bias, we used the capture and detection antibodies from this kit to carry out Western blotting on patient/HC serum (n=11) **(B)** (t-test p<0.05). As can be seen, all the recognised CRP species are >70 kDa, as such limiting detection to pentameric and tetrameric forms. To confirm the presence of monomeric CRP in these samples, we carried out a monomeric specific ELISA **(C)** with CRP-8, which showed significantly higher levels of mCRP in RA compared to controls (**C**, p<0.05 n=11). Western blotting identified bands ~25–35 kDa, consistent with CRP’s monomeric size **(D)**. This strongly suggests that clinical assays are invisible to mCRP.

### mCRP levels positively correlate with anti-CCP antibody level

Next, demographic and clinical data were interrogated to identify potential correlations between clinical measures and mCRP levels. A modest, though significant correlation was identified between mCRP levels and anti-CCP antibody levels in serum (r=0.39, p 0.02) (**Figure 3a**).Following this, to identify potential clinical characteristics of mCRP-dominant and pCRP-dominant patients, they were subdivided into four categories with low and high mCRP/pCRP thresholds set at three standard deviations below and above the HC mean, respectively: (1) low mCRP/low pCRP, (2) low mCRP/high pCRP, (3) high mCRP/low pCRP, and (4) high mCRP/high pCRP. There was a trend for higher levels of anti-CCP in patients with predominant mCRP; however, no significant association was seen for rheumatoid factor (RF) (it is of note, however, that the three highest levels for RF were in the high-mCRP patients) (**Figure 3b**). Further analysis of clinical testing identified significantly higher white cell counts in patients with a low mCRP/high pCRP profile compared to low pCRP/low pCRP, which was recapitulated in neutrophil levels (**Figure 3c**). Correlations with pCRP were significant against white blood cell count across both cohorts; however, no correlation was seen for mCRP (**Figure 3d**). Furthermore, this correlation remained significant regardless of levels of mCRP in the patient blood (**Figure 3e**), confirming this as a function of pCRP alone. This is in line with pCRP driving white cell expansion and influencing neutrophil behaviour whereas mCRP cannot (16, 17). A subanalysis using patients previously characterised as high or normal CRP identified a similar trend, suggesting that any changes are not due to just levels of CRP (**Supplementary Figure 3**).

**Figure 3:**
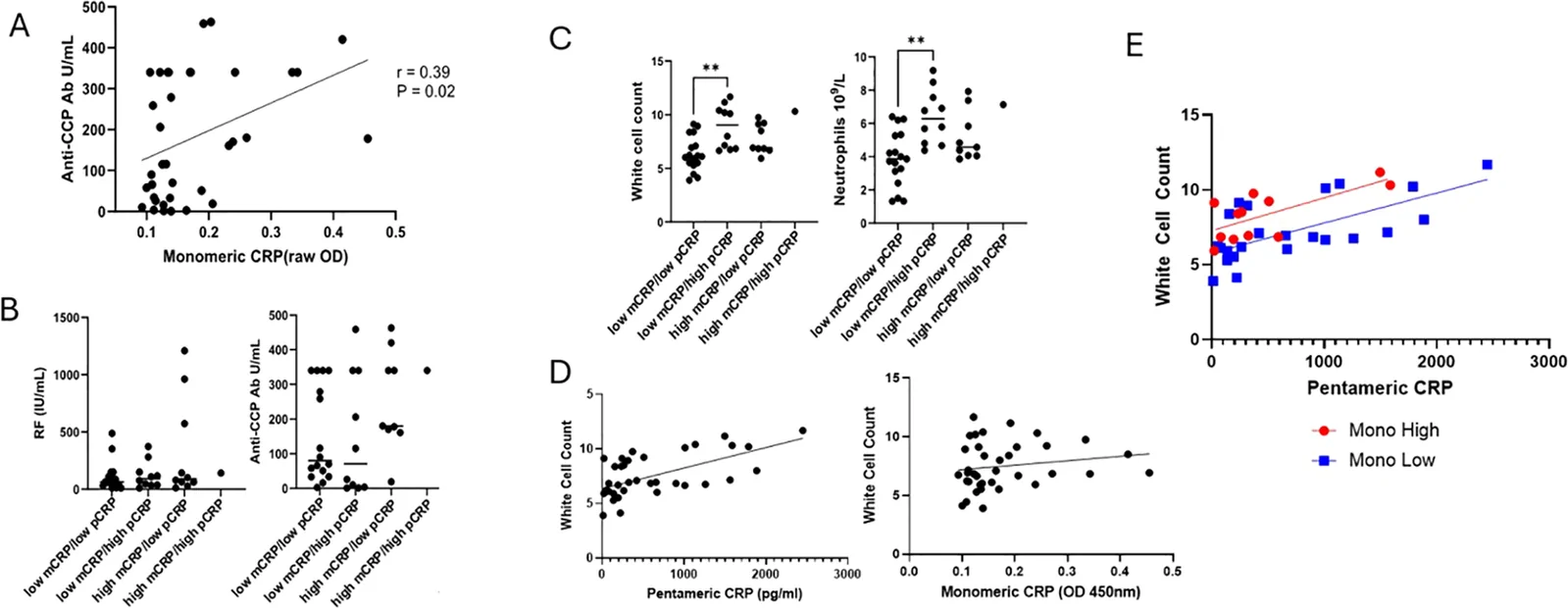
mCRP is linked to anti-CCP antibody levels. To assess for clinical utility, we split patients into high and low mCRP and pCRP based on a cutoff derived from 3×SD from the mean for each group. Interestingly, when assessing antibody positivity **(A)**, a clear association was seen with higher levels in patients with primarily mCRP with relatively lower amounts of pCRP, with both RF and anti-CCP. A correlation analysis **(B)** showed a moderate correlation between mCRP levels and anti-CCP across RA patients which was statistically significant (r=0.39, p=0.02, n=39); this was not possible for RF which showed no significance. To further assess potential functional/clinical effects, we assessed white blood cell count and neutrophil count, showing that both were associated with high pCRP rather than mCRP, as per literature **(C)**. Correlation of the pCRP and mCRP levels with white blood cells showed a significant correlation for pCRP (p=0.001 r=0.6) but not for mCRP **(D)**. Furthermore, splitting these to look at mCRP high and mCRP low showed that pCRP correlated well regardless of mCRP status (high mCRP p=0.011, r=0.4895, low mCRP p=0.0002, r= 0.4571). ** is <0.01.

### Dissociation of CRP is not CaCl_2_ or NaCl_2_ dependent; however, CRP in RA serum is more sensitive to shear stress compared to healthy control

To explore potential mechanisms by which pCRP may degrade to mCRP in serum from patients and controls, dissociation experiments were performed in the presence of calcium and salt gradients at various temperatures alongside chelation. Salt gradients aimed to reduce ionic interactions, whereas temperature was to increase energy in the system. Chelation targeted the calcium ions between monomers. Most patients with RA had elevated levels of mCRP which were absent in the healthy control serum (**Supplementary Figure 1**); however, neither chelation nor salt or calcium gradients influenced the levels of mCRP (**Supplementary Figure 1A**), suggesting that in-solution dissociation is neither a function of stability nor charge of metal ions. To further study mCRP dissociation, we applied sheer force through centrifugal concentration. Sera were exposed to high stress through centrifugal concentration using a membrane of 50 kDa (**Supplementary Figure 1B**). This method identified potential instability in the CRP from patients with RA compared to HCs. The fractions above and below the membrane were assayed for structures of CRP. Interestingly, for most patients, mCRP was detected in the fraction taken from above the membrane in contrast to the fraction below. In contrast, HC identified no mCRP after centrifugation (**Supplementary Figure 1C**). Levels of mCRP were not proportional to pCRP identified, suggesting that this may not be the only method of dissociation; however, intrinsic instability may exist in serum CRP in patients with RA. It is also possible that this mCRP is complexed with something forcing it to be retained above the membrane in RA, which is not the case for HC.

### Body temperature (37 °C) has a unique protein dynamic favouring dissociation

To study the dynamics of unfolding, we utilised molecular simulations at different temperatures. Specifically, the protein was modelled at 30 °C, 37 °C, and 50 °C to allow for identification of rapid changes (30 °C), body temperature-specific changes (37 °C), and a higher temperature to increase energy in the system to encourage potential unfolding.

Initial measurements of global movement or unfolding (by root mean square deviation (RMSD) or radius of gyration (R_G_)) showed that the most movement and the most unfolded structures were in the 37 °C simulation (**Figure 4A**, red line). This suggests that the 37 °C structure explores more conformations than either the 30 °C or 50 °C simulations. To further examine this, we conducted principal component analysis of the three simulations combined, identifying structures specific to certain temperatures, and the path that each simulation took through these structures (**Figure 4B**). The first three PCs accounted for 65% of total variance, whereas a K-means clustering showed that using six clusters was sufficient to capture the major conformations explored. All simulations began in the black structure and passed through the red structure; however, at 30 °C, these two structures accounted for ~60% of the simulation, before moving into a similar structure (in blue). In the PCA plot coloured by time, we demonstrate a function by which the simulation transitions back and forth amongst these three clusters. The 50 °C structure also demonstrates altered kinetics, beginning in the same dominant structures from 30 °C (black, red, blue) before finding a unique structure in pink, and transitioning back and forth from this through to structures early in the simulation. In stark contrast, the 37 °C simulation spends the least time in the black structure and transitions to two unique structures, in green and cyan. These both exist in a PCA space neither the 30 °C nor the 50 °C simulations explore (**Figure 4B**). Furthermore, analysing the RMSD and R_G_ of these structures identifies that the structure unique to 50 °C has the smallest R_G_ and low RMSD (**Figure 4C**), suggesting that this is a compact circular shape, whereas the unique structures in the 37 °C protein (green and cyan) represent the largest change from the starting position and are the least compact. This suggests that 37 °C may represent a unique opportunity for CRP to open and dissociate, whereas the back and forth transitions of the other simulations may represent a kind of molecular breathing, repeatedly transitioning between the same states.

**Figure 4:**
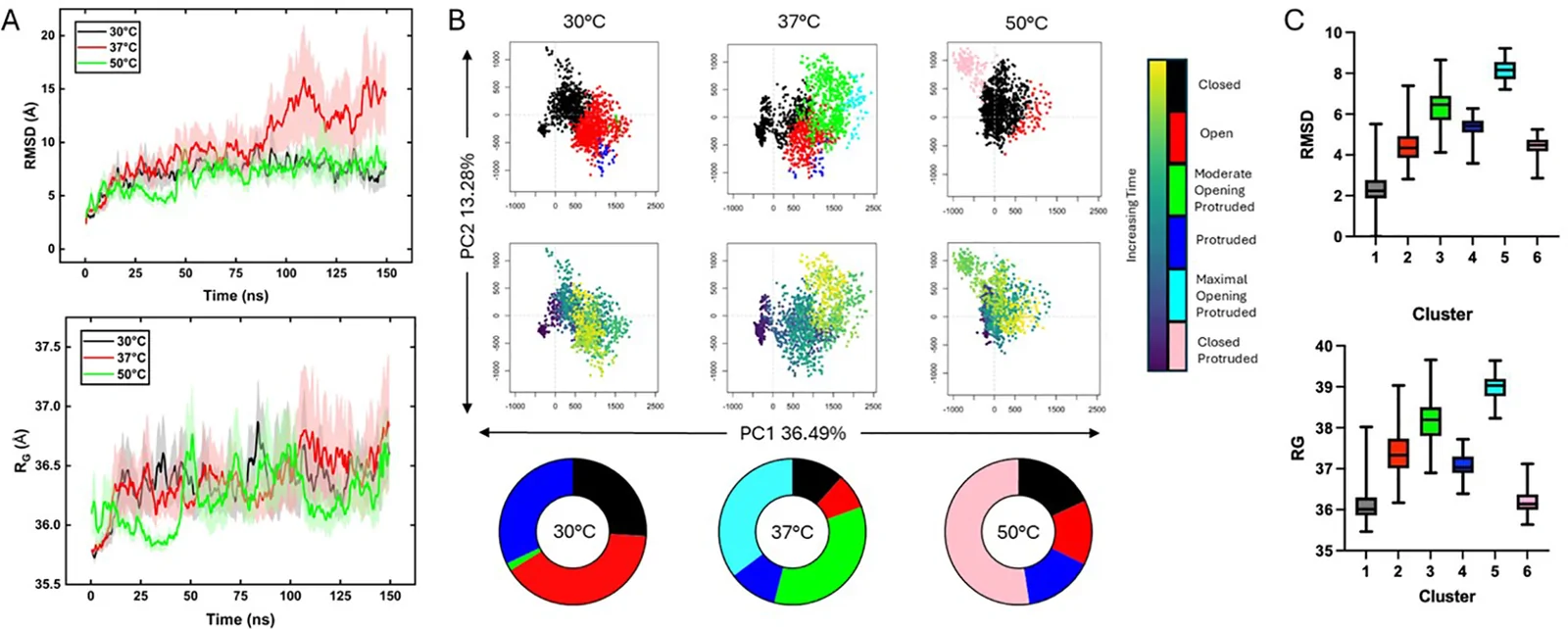
Molecular simulations suggest a Goldilocks effect. Simulations of pCRP at 30°, 37 °C, and 50 °C were carried out. Measurement of RMSD and Rg **(A)** showed higher RMSD values in the simulation at 310 K (37 °C) compared to either other temperature suggesting increased movement, whereas the average Rg was also higher, suggesting a less compact molecule. Structures were aligned and interrogated by PCA to identify structures specific to temperatures **(B)**. Whilst the 30 °C and 50 °C simulations occupied similar PCA space, the 37 °C simulation explored unique space; quantifying the relative abundance of these structures showed a predominance for the maximal opening protruded structure (cyan) and the moderate open protruding structure (green) at 37 °C, whereas the closed structure (pink) was most prominent at 50 °C. This difference in structure was associated with a different pathway throughout the simulation with the lower temperature fluctuating across a midpoint (viridis scale PCA) whereas the highest-temperature simulation moved back and forth to a central space. The 37 °C trajectory was the only one to show a movement to end in a novel PCA space. **(C)** Measuring the RMSD in the identified different structures showed the highest RMSD structures were within the 37 °C simulation, and associated with a higher Rg.

We then used a dynamic cross correlation matrix analysis (DCCM) to identify potential intra-protein regulatory networks (**Figure 5**). Initially, we identified areas of altered correlation in the 37 °C simulation with respect to either the 30 °C or 50 °C simulations (**Figure 5A**). An altered correlation was seen for associations between PROA (protein chain A), PROB (protein chain B), and PROE (protein chain E), representing the first, second, and fifth monomers. When plotting the direction of the altered correlation (**Figure 5B**), we identified loss of positive correlation between PROA and PROB, whereas a loss of negative association between PROB and PROE was also seen, suggesting that these correlations may contribute to the stable closed structures at both 30 °C and 50 °C, respectively. Finally, plotting these out on the structure itself (**Figure 5C**) shows that the altered correlation of PROE to PROA is at the interface of the two domains, whereas the PROB/PROA alterations are at their interface. Interestingly, PROE and PROB also have altered correlation to the third monomer (PROC (protein chain C), although this is far less. Finally, we aligned the midpoints of the clusters representing the closed form (pink) and the most open form (cyan), and as can be seen, opening appears to be a function in multiple axes, with the monomers moving out of alignment horizontally rather than directly out of the circular form (**Figure 5D**).

**Figure 5:**
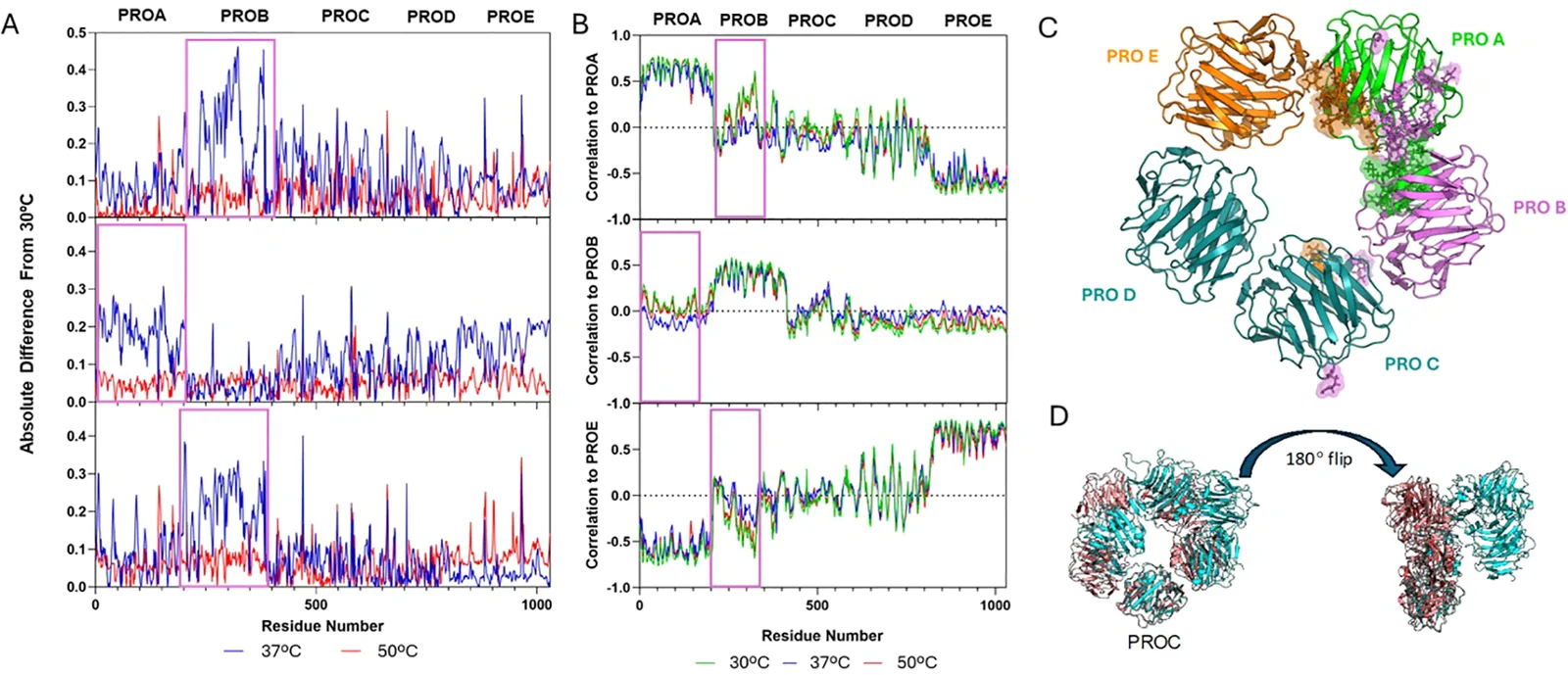
Simulations reveal structures with differential exposure. Using correlation matrices, we calculated the correlations of each atom to every other atom, every other 0.1 ns across the simulations at different temperatures to highlight correlative networks differentially regulated by temperature. We then identified areas of significant change by calculating absolute correlative change from baseline (30 °C), which highlighted regions in PROA, PROB, and PROE with correlative change unique to the 37 °C simulation. **(A)** We then plotted these separately with the regions of interest and showed loss of significant correlative effect for PROA vs PROB, with a similar loss **(B)** in PROB vs PROA, though not in matching regions. Furthermore, PROE lost the negative correlation with PROB, showing that PROB was a site of significant structural interest in the molecule. Plotting those sites on the molecule **(C)** showed a potential long distance network of correlation, orange peptides demonstrating changes with PROE correlation, pink showing correlations with PROB changed, and the green modifications showing sites in PROB altered by PROA movement at 37 °C. Aligning the closed and maximally open frame by PROC, highlighted the movement in multiple directions for opening, which involved the sites plotted in **(D)**.

These simulation data, demonstrating a goldilocks zone for the protein and a molecular breathing movement, suggest that at the body temperature, CRP may have a greater tendency to separate to monomers.

### Flow cytometry identifies alterations in monocyte surface markers

Having shown circulating mCRP, we further investigated evidence of its activity in circulation. Using spectral flow cytometry, we investigated the myeloid compartment of PBMCs isolated from patients with low mCRP (group 1; n=4) compared to those with high mCRP (group 2; n=4) (**Figure 6A**). We found that mononuclear phagocytes (MNPs) from our high mCRP group 2 had significantly lower mean fluorescence intensity (MFI) expression of CD11c, a marker typically associated with dendritic cells (DCs) and mediates key immunological functions such as cell adhesion, migration, and phagocytosis (18). This decrease seen in high mCRP patients is in line with current literature showing that higher levels of CRP can impair DC maturation and function (19). Our data suggests that high mCPR alone in the presence of low pCRP may be involved in this impaired DC maturation; however, we acknowledge that as no investigation of circulating myeloid phenotype was carried out in patients with high pCRP, it is not possible to know if the alterations in cell phenotype are due to mCRP alone or are also observed in patients with high pCRP.

**Figure 6:**
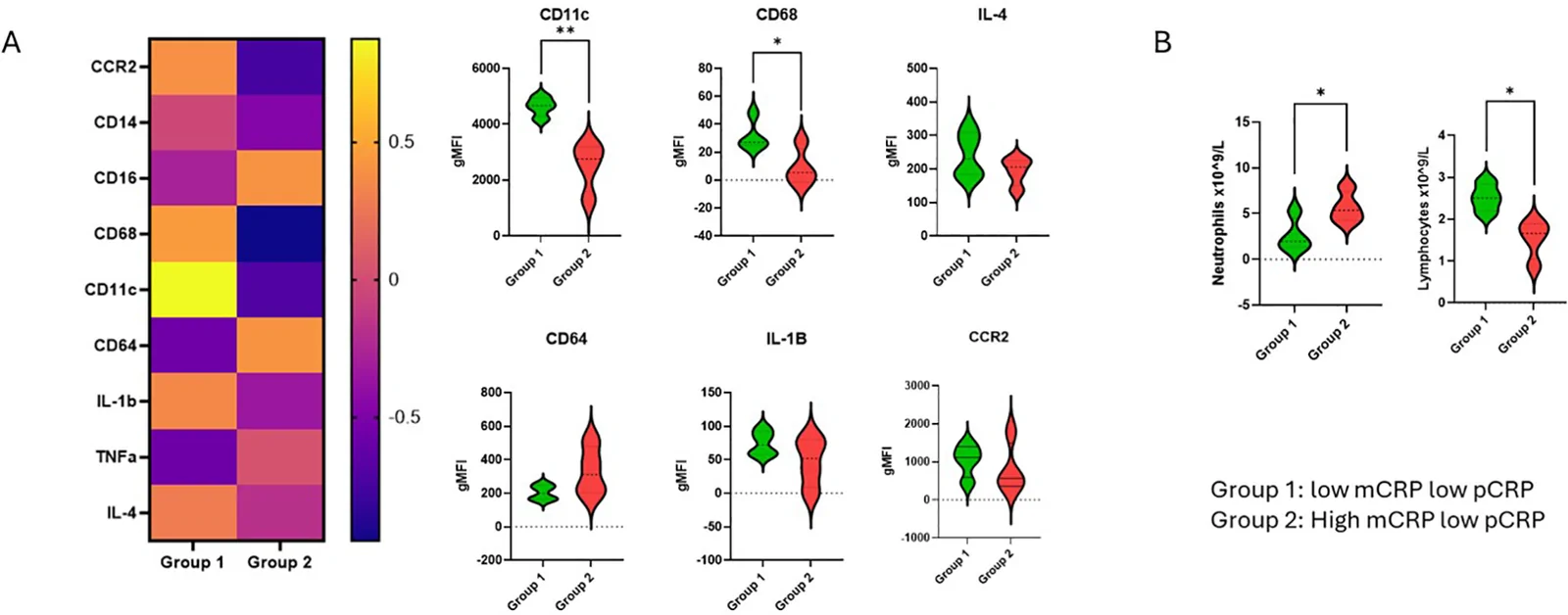
Cellular changes associated with mCRP. We conducted high-dimensional flow cytometry on the PBMCs from a subset of RA patients with low pCRP and low mCRP (group 1, n=4) and compared them to patients with low pCRP and high mCRP (group 2, n=4) to assess if there were changes in profile. Investigating specifically the myeloid compartment, we found small phenotypical changes in mononuclear phagocytes (HLA-DR^+^CD3^-^CD19^-^CD56^-^). **(A)** Heatmap representing changes in mean fluorescence intensity (MFI) of selected markers of mononuclear phagocyte phenotype and function between groups and associated violin plot representation. Specifically, there was a significant decrease in the mean fluorescence intensity (MFI) of CD11c (integrin alpha X) and CD68 in addition to some non-significant trends. This is caveated by low numbers in the groups, and data that showed trends which were non-significant are likely due to the low numbers in the groups. Finally, in **(B)** we plot the clinically derived numbers of cells, showing increased neutrophils but lower levels of white blood cells across group 2 compared to group 1.​ A p value of <0.05 was taken as the threshold of significance with graphical representation as; p < 0.05 = *, p < 0.01 = **.

We also noted that there was a significant decrease in the expression of CD68, typically a marker of macrophage differentiation in MNPs. Some subtle trends in monocyte phenotype were noted, particularly with markers traditionally used to separate monocytes into the well-established classical, intermediate, and non-classical subsets with high mCRP MNPs appearing to lean towards a more non-classical monocyte phenotype with a potential elevated expression of CD16 and a lower expression of CD14 and CCR2 compared to group 1. This is interesting as, in literature, non-classical monocytes are mediators of damage in RA (16, 25). However, these data presented here are limited by low numbers in the groups, and data showed trends which were non-significant likely due to the low numbers in the groups, making it difficult to draw further conclusions.

Evaluation of collected clinical measurements revealed that whilst there were increased neutrophils in the high mCRP group, there were decreased lymphocyte numbers (**Figure 6B**). This would classically indicate that there is a significant ongoing acute inflammatory response usually triggered by severe stress, infection, or chronic inflammatory settings such as autoimmune disease.

## Conclusions

We demonstrate that mCRP exists in the blood of people with RA at a greater level than healthy controls. This appears invisible to clinical assays, suggesting that mCRP may provide new insights into novel mechanisms of pathogenesis in RA patients. Further to this, we also supply the first comprehensive simulation of pCRP investigating potential dissociation, highlighting that even within increased energy to allow the exploration of energetically unfavourable states, pCRP remains stable, with the greatest instability at body temperature. This suggests that dissociation takes place in a “goldilocks” zone of conditions at 37 °C. Finally, we showed a novel correlation that levels of mCRP correlate significantly with anti-CCP levels, which may suggest that mCRP could be linked to production by an as yet undefined mechanism.

Clinical associations to levels of mCRP and pCRP also corroborated findings in literature (20), which show that pCRP drive white blood cell expansion, particularly neutrophil populations. Our cohort is small; therefore, further testing is required. Conversely, monomeric CRP levels correlate with anti-CCP antibodies, suggesting either a causal or correlational relationship between them. Coupled with the dissociation data, these findings indicate that novel posttranslational modification (PTMs) of CRP may exacerbate pCRP dissociation into mCRP, serving to increase citrullinated-antigen availability and amplify anti-CCP Ab production through greater antigenic stimulation or through mCRP-driven IL-6 production, stimulating Ab secretion from plasmablasts (21). Interestingly, abnormal PTMs are known to contribute to loss of tolerance against self-peptides within RA (22) and a change in protein function ranging from citrullination and carbamylation and glycosylation. CRP is susceptible to these PTMs (23), and this may in some way explain why enhanced pCRP dissociation in RA serum is seen. Additionally, Stein, Cassol, and Moresco (24) demonstrated that cyanate presence lowers CRP detectability by 61%-72%, suggesting that increased PTMs could also contribute to decreased CRP levels in RA patients (as cyanate is directly linked to carbamylation).

Molecular dynamics analysis revealed a body-temperature-driven structural opening and protrusion of pCRP, which likely promotes dissociation, suggesting its emergence as a natural consequence of pentameric flexibility. This accounts for the cluster distribution across temperatures as 37 °C supplies enough energy to drive expansion without enabling recoil. Opening and protrusion of pCRP in a lower energy state confirm the thermodynamic favourability of dissociation, whereas increased temperatures amplified the “recoil” effect ending with a tighter final confirmation than the start. This likely offers a mechanistic perspective on how mCRP formation during acute inflammation is inhibited, leading to the accumulation of pCRP that can be measured in blood (4), characteristic of the acute response. Interestingly, with RA flares often associated with fevers, this mechanism potentially forces CRP to remain pentameric and thus again control function.

Most importantly, it was shown by Western blotting that validated ELISAs may be blind to mCRP, with our commercial ELISA corroborating clinical results but not detecting mCRP. Further assaying with an mCRP-specific antibody identified mCRP in the same samples; however, as previously discussed, the mCRP ELISA requires significant validation for sensitivity and specificity before widespread use going forward. Further to this, Western blots under standard and dissociative conditions reveal different amounts of novel CRP intermediate structural compounds in both healthy and RA serum, which may imply that intermediate structures are more stable than previously suggested (4). This must in our work be taken with caution as our healthy controls are significantly younger than the RA patients; as such, we cannot rule out the effect of age on CRP using our work. Our data instead supports a stepwise dissociation of pCRP into a monomer with all intermediates stable and detectable in serum. The presence in serum also suggests that if dissociation requires a membrane surface that these intermediates are released from the membrane to further dissociate, or alternatively that the membrane is not required and dissociation is taking place in solution. Further research is required to confirm this as without being able to trigger dissociation in our own work (using calcium, salt, and chelating agents), it is hard to monitor the rate of dissociation on a monomer-by-monomer basis.

In conclusion, our study suggests that mCRP may be a significant marker for RA disease activity and may also play a role in the pathogenesis of Rheumatoid Arthritis.

## Supplementary MD analysis

All simulations were combined into a single trajectory and analysed together in order to identify potential structures unique to one condition. Initially, PCA analysis of all MD simulations revealed six distinct structural conformations for pCRP with the first 3 PCs accounting for 65% of all variance seen across simulations. Cluster overlap was assessed by comparing median RMSD and R_G_ across clusters demonstrating structural divergence and protein compactness where clear stratification is seen with both RMSD and R_G_ (**Figure 4**). Similar RMSD values can be seen across cluster two and six (median 4.34 Å and 4.48 Å) however these clusters demonstrate distinct R_G_ distributions (median 37.33 Å and 36.14 Å) – signifying that, despite similar degrees of atomic deviation, these clusters represent conformations with different spatial compactness. These findings support the identification of six distinct pCRP conformational states.

Several of these clusters were, however, temperature specific. Cluster six was only identified at 50 °C, whilst clusters three and five were predominantly found at 37 °C. No structures were restricted to the 30 °C simulation, however, it did encompass the majority of cluster two events (**Figure 4**). Using the cluster mid-points as representative frames from each cluster revealed a range of opening stages for CRP across the simulations (**Figure 4**). It was observed that pCRP transitions between three primary states (closed/open/protruded) and three hybrid states (moderate/maximal opening protruded/closed-protruded state) encompassing changes in PROA-PROE separation distance and protrusion of PROA/PROB/PROC. We also identified the pronounced structural divergence between the most closed (cluster six) and open-protruded (cluster five) states where a 34.7 Å outward displacement of PROA and a 32.6 Å increase in PROA-PROE distance can be seen (**Supplementary Figures 1**, **2**). Unexpectedly, the most protruded form (cluster five) exists within the 37 °C simulation whilst the most compact (cluster six) is from the highest energy simulation (50 °C).

To examine the temporal trajectory of pCRP’s conformational transitions, PCAs on individual simulation repeats utilizing the previously defined clustering was performed. These have been visualised in **Figure 4** which includes both colouring by cluster and by time. Even within the individual clusters, temperature dependent differences can be seen, for example, those structures in cluster 2 originating from the 37 °C simulation represent a unique group which sit mainly below the X axis in PC2. This highlights the increase in divergent trajectory in the 37 °C simulation. Similarly, examining for time, it can be seen that 37 °C simulation demonstrates a transition across a greater conformational space than either of the other simulations, with a more linear process, finishing in a distinct space compared to the initial simulation starting point. In contrast, both the 30 °C and the 50 °C simulations appear to explore conformations before returning to their starting structure. Whilst all the simulations show some level of recoil through a stable intermediary state, this state for the 37 °C sits in a distal space to its original structure. This trajectory has illustrated that pCRP undergoes a mechanism of molecular breathing, defined by an early expansion phase followed by a recoil stabilisation phase. During the former, PROA and PROE are subjected to horizontal separation accompanied by a protrusion of PROA/PROB/PROC away from remaining subunits. The subsequent recoil stabilisation phase is characterised by a vertical increase in PROA-PROE distance coupled with partial retraction of protruded subunits, resulting in a final conformation that is both more open and protruded than the initial closed state. The duration of these two general movements is temperature dependent, at 30 °C, the expansion and recoil stabilisation phases are equally distributed between early and late-phase trajectories with 50% of the open conformation appearing at this temperature. However, the expansion phase at 37 °C extends into its late phase trajectory where maximal opening and protrusion can be seen, accounting for 97% and 100% of moderate/maximal opening protruded conformations respectively (clusters five and six). Finally, recoil stabilisation beginning in the early-stage trajectory can be seen at 50 °C. This trajectory also features a recoil of its opening mechanism which is not seen in any other simulation, resulting in a final confirmation that is both tightly closed and protruded.

Further to this we conducted DCCM analysis to identify potential hot-spots of movement within the protein which may drive dissociation. Through plotting the absolute difference from the 30 °C trajectory compared to the 37 °C and 50 °C trajectories it is possible to identify the amino acids with disrupted long distance correlations. Interestingly, PROA, B and E all showed significant changes in their long distance associations with PROA and PROB altering in correlation to each other whilst PROE also altered association with PROE (**Figure 4**). Further to this, plotting out the direction shows a loss of correlation between PROA and PROB, whilst the correlation of PROB to PROE shows a gain in correlation at 37 °C. Plotting the top 20 amino acids with the most changed correlation values (**Figure 4**) identified a number of sites of interest, primarily at the interface of PROA and PROB, whilst some sites in PROC showed altered values potentially showing a hinge region forming, these are shown with the associated domain colouring (PROE correlation changes are coloured orange etc).

## Data Availability

The raw data supporting the conclusions of this article will be made available by the authors under reasonable request.

## Supplementary material

Supplementary Figure 1 Stability and Dissociation is Altered in RA Serum. Initial experiments to dissociate pCRP in the fluid phase showed that neither CaCl2 nor NaCl gradients alone could induce dissociation in healthy control serum (HC1) or RA Serum (RA6,RA7). Centrifugal concentration **(B)** showed no mCRP above the membrane for HC **(C)** whilst the majority of RA samples showed CRP retained above the membrane’s cut off, but separated by gel to be monomeric suggesting the increased sheer force drives further dissociation from the pentameric fraction. Please note these membranes were cut at the 38kDa marker and separately probed and exposed.

Supplementary Figure 2 PROA protrusion distance determination. **(A)** First residue of PROA in cluster six structure (pink) was selected and distance to the same residue in cluster five structure (cyan) was measured. **(B)** Lys13 was selected as starting point for calculation due to its overlapping nature in both cluster five (cyan) structure and cluster six (pink) structure. Next, distance was calculated to the first residue in PROA for each structure.

Supplementary Figure 3 nCRP and hCRP did not explain monomeric/pentameric splits. We separated the patients into those with high CRP (hCRP) and normal levels of CRP (nCRP)as previously described. Whilst this split remained using the clinical assay **(A)** it was not shown to be significantly different in monomeric CRP **(B)**. A difference was seen in the strength of correlation for the hCRP vs anti-CCP levels, however, a similar trend though non-significant, existed for the nCRP cohort the correlative effect of anti-CCP with mCRP held for hCRP patients when split to hCRP and nCRP (r=0.29, p=0.04), whilst nCRP was a strong trend (p=0.11) with a lower R squared value (r=0.126).
