# Supplementary material for: Monomeric CRP is prevalent in the blood of patients with rheumatoid arthritis and correlates with clinically relevant markers
Source: Front Immunol. 2026 Sep 18;17:1925147. doi: 10.3389/fimmu.2026.1925147 (PMC13630960; doi:10.3389/fimmu.2026.1925147)
Supplement: Supplementary file 1 [file DataSheet1.pdf]

Supplementary Table 1: Patient Joint Erosions. This table shows the high and low pentameric/ monomeric CRP levels and the joint erosions, grade of damage and which joints. No difference was seen for high mCRP vs low mCRP.

Study recruit	Mono class	Penta class	Number of joints with SH grade 1	Number of joints with SH grade 2	Number of joints with SH grade 3	Number of joints with effusion	Which joints?	Number of joints with erosions	Which joints have erosions	Number of joints with osteophytes	Which joints?	Tendon abnormalities (how many, which tendons)	TJC/28	SJC/28	GVAS/ 100	HAQ
4	High	High	0	6	2	0		0		0			7	0	80	8
11	High	Low	6	3	10	0		20	L. Radial, IC, Ulnar, MCP1, MCP2, MCP5, PIP1, PIP2, PIP3, PIP4. L. Radial, IC, MCP1, MCP2, MCP4, PIP1, PIP2, PIP3, PIP4, PIP5.	1	R. MCP5.		20	16	100	6
22	High	Low	4	20	1	0		2	L. MCP5. R. PIP5	0			22	8	70	10
26	High	Low	1	1	0	0		0		0			7	2	55	4
27	High	Low	12	0	0	1	L. PIP1.	17	L. MCP2, MCP3, MCP5, PIP3, PIP5. R. Radial, IC, Ulnar, MCP2, MCP3, MCP4, MCP5, PIP1, PIP2, PIP3, PIP4, PIP5.	0			5	2	55	5
29	High	Low	2	11		0		1	IC				12	10	65	6 to 7
32	High	Low	2	4	0	1	R. Radial.	0		0			2	2	30	8
35	High	Low	1	0	2	0		7	L. MCP2 and PIP2 R. MCP2, PIP1, PIP2, PIP3, PIP5.	5	L. PIP2 and PIP5. R. PIP1, PIP2 and PIP5.		4	3	10	1
36	High	Low	4	8	0	0		8	L. Radial, IC, Ulnar, MCP3, MCP5. R. MCP1, MCP2, MCP4	0			1	4	60	5
40	High	Low	7	0	5	0		3	MTP2, MTP2, MTP3	0						10
54	High	Low		8	0	0		16	L. Radial, IC, Ulnar, MCP1, PIP1, PIP2, PIP4. R. Radial, IC, Ulnar, MCP1, MCP2, MCP3, MCP4, MCP5, PIP1.	0			0	0	15	
12	Low	High	1	1	0	1	R. PIP1.	8	L. Radial, IC, Ulnar. R. Radial, IC, Ulnar, MCP5, PIP5	2	R. PIP1 and PIP3.		13	4	70	7
23	Low	High	1	2	1	0		2	R. IC, Ulnar.	1	R. PIP1.		1	3		
28	Low	High	1	11	3	0		15	L. MCP1, MCP2, MCP3, MCP5, PIP2, PIP3. R. Radial, IC, Ulnar, MCP2, MCP3, MCP4, MCP5, PIP4, PIP5.	7	L. MCP1, PIP2, PIP3 and PIP5. R. PIP1, PIP4 and PIP5.		16	3	10	1
30	Low	High	3	2	7	0		2	L. PIP1. R. IC.	0			5	9	40	3.5
33	Low	High	2	5	3	0		0					6	4	100	10
38	Low	High	2	1	2	0		4	L. PIP2 and PIP3. R. IC and Ulnar	0			13	5	60	8
39	Low	High	4	5	6	0		6	L. PIP1, PIP3, PIP5. R. IC, MCP1 and MCP5.	0		0	22	15	75	7.5
45	Low	High	0	1	1	0		6	Radial, IC, Ulnar, Radial, IC, Ulnar	0			4	3	70	6
48	Low	High	2	2	6	0		9	L. Radial, IC, Ulnar, MCP3. R. Radial, Ulnar, MCP3, MCP4, MCP5.	1	L. MCP3.		8	5	80	8
50	Low	High	2	0	0	0		2	R. Ulnar and PIP2.	1	L. PIP2.		0	0	40	3
3	Low	Low	6	1	0	2	L. MCP3 and R. radial.	2	L. IC and R. Ulnar.	3	L. PIP1 and PIP2. R. PIP5.		15	2	75	8
7	Low	Low	2	10	0	1	R. MCP5.	18	L. Radial, IC, Ulnar, MCP1, MCP2, MCP3, PIP2, PIP3, PIP4. R. Radial, IC, Ulnar, MCP1, MCP2, MCP3, PIP1, PIP2, PIP4	0			12	9	75	7.5
8	Low	Low	2	2	0	4	L. MCP2 and MCP4. R. Radial and MCP2.	1	R. Ulnar.	1	R. PIP1.	0	2	1	20	3
13	Low	Low														1
16	Low	Low	2	3	0	0		0		0			18	1		9
19	Low	Low	3	1	0	0		2	R. Ulnar, MCP2.	1	L. MCP2.		12	2	70	7
21	Low	Low	4	15	4	0		21	L. Radial, IC, Ulnar, MCP2, MCP3, MCP4, MCP5, PIP1, PIP2, PIP3, PIP4, PIP5. R. Radial, IC, Ulnar, MCP3, MCP5, PIP1, PIP2, PIP3, PIP5.	3	L. MCP2 and MCP3. R. MCP2.	0	5	6	10	8
24	Low	Low	4	7	4	0		16	L. Radial, IC, Ulnar, MCP1, MCP2, MCP3, MCP4, MCP5. R. Radial, IC, Ulnar, MCP2, MCP3, MCP4, MCP5 and PIP2.	0		0			10	
25	Low	Low	5	6	2	0		3	IC, MTP1 MTP5	2		Fluid ECC?	1	4	80	9

31	Low	Low	0	4	4	2	R. MCP5 and PIP5.	1	L. MCP5	0			20	13	80	8
34	Low	Low	4	3	4	0		8	L. MCP2, MCP3. R. Radial, IC, Ulnar, MCP2, MCP5, PIP1.	1	R. PIP1.		16	3	70	2
37	Low	Low	3	2	3	0		0		0			7	10	90	5
43	Low	Low	1	2	1	0		0		0			8	2	60	8
44	Low	Low	7	6	2	1	L. MCP4 Grade 3	14	L. Radial, Ulnar, MCP1, MCP2, MCP3, MCP4, MCP5. R. Radial, IC, Ulnar, MCP1, MCP2, MCP3, MCP5.	0		Extensor Carpi Ulnaris tenosynovitis	28	3	50	4
46	Low	Low	0	8	15	1	R. PIP1 (Grade 2?)	20	L. Radial, IC, Ulnar, MCP1, MCP2, MCP3, MCP4, MCP5, PIP1, PIP2, PIP3 ,PIP4. R. MCP1, MCP2, MCP3, MCP4, MCP5, PIP2, PIP3, PIP4.	0			1	11	0	3
47	Low	Low	3	4	0	3	L. MCP2. R. Radial and PIP1.	1	R. PIP3	0			18	4	35	3
53	Low	Low		7		1	L. MCP3.	15	L. Radial, IC, MCP1, MCP3, MCP5, PIP1, PIP2. R. Radial, IC, Ulnar, MCP1, MCP2, PIP2, PIP3, PIP5.	2	R. PIP2 and PIP3.		5	2	70	
