# Supplementary material for: Monomeric CRP is prevalent in the blood of patients with rheumatoid arthritis and correlates with clinically relevant markers
Source: Front Immunol. 2026 Sep 18;17:1925147. doi: 10.3389/fimmu.2026.1925147 (PMC13630960; doi:10.3389/fimmu.2026.1925147)
Supplement: Supplementary file 2 [file DataSheet2.pdf]

Supplementary MD Analysis

All simulations were combined into a single trajectory and analysed together in order to identify potential structures unique to one condition. Initially, PCA analysis of all MD simulations revealed six distinct structural conformations for pCRP with the first 3 PCs accounting for 65% of all variance seen across simulations. Cluster overlap was assessed by comparing median RMSD and R_G across clusters demonstrating structural divergence and protein compactness where clear stratification is seen with both RMSD and R_G (**Figure 4**). Similar RMSD values can be seen across cluster two and six (median 4.34 Å and 4.48 Å) however these clusters demonstrate distinct R_G distributions (median 37.33 Å and 36.14 Å) – signifying that, despite similar degrees of atomic deviation, these clusters represent conformations with different spatial compactness. These findings support the identification of six distinct pCRP conformational states.

Several of these clusters were, however, temperature specific. Cluster six was only identified at 50°C, whilst clusters three and five were predominantly found at 37°C. No structures were restricted to the 30°C simulation, however, it did encompass the majority of cluster two events (**Figure 4**). Using the cluster mid-points as representative frames from each cluster revealed a range of opening stages for CRP across the simulations (**Figure 4**). It was observed that pCRP transitions between three primary states (closed/open/protruded) and three hybrid states (moderate/maximal opening protruded/closed-protruded state) encompassing changes in PROA-PROE separation distance and protrusion of PROA/PROB/PROC. We also identified the pronounced structural divergence between the most closed (cluster six) and open-protruded (cluster five) states where a 34.7 Å outward displacement of PROA and a 32.6 Å increase in PROA-PROE distance can be seen (Supplementary figures 1 and 2). Unexpectedly, the most protruded form (cluster five) exists within the 37°C simulation whilst the most compact (cluster six) is from the highest energy simulation (50°C).

To examine the temporal trajectory of pCRP's conformational transitions, PCAs on individual simulation repeats utilizing the previously defined clustering was performed. These have been visualised in figure 4 which includes both colouring by cluster and by time. Even within the individual clusters, temperature dependent differences can be seen, for example, those structures in cluster 2 originating from the 37°C simulation represent a unique group which sit mainly below the X axis in PC2. This highlights the increase in divergent trajectory in the 37°C simulation. Similarly, examining for time, it can be seen that 37°C simulation demonstrates a transition across a greater conformational space than either of the other simulations, with a more linear process, finishing in a distinct space compared to the initial simulation starting point. In contrast, both the 30°C and the 50°C simulations appear to explore conformations before returning to their starting structure. Whilst all the simulations show some level of recoil through a stable intermediary state, this state for the 37°C sits in a distal space to its original structure. This trajectory has illustrated that pCRP undergoes a mechanism of molecular breathing, defined by an early expansion phase followed by a recoil stabilization phase. During the former, PROA and PROE are subjected to horizontal separation accompanied by a protrusion of PROA/PROB/PROC away from remaining subunits. The subsequent recoil stabilization phase is characterized by a vertical increase in PROA-PROE distance coupled with partial retraction of protruded subunits, resulting in a final conformation that is both more open and protruded than the initial closed state. The duration of these two general movements is

temperature dependent, at 30°C, the expansion and recoil stabilization phases are equally distributed between early and late-phase trajectories with 50% of the open conformation appearing at this temperature. However, the expansion phase at 37°C extends into its late phase trajectory where maximal opening and protrusion can be seen, accounting for 97% and 100% of moderate/maximal opening protruded conformations respectively (clusters five and six). Finally, recoil stabilization beginning in the early-stage trajectory can be seen at 50°C. This trajectory also features a recoil of its opening mechanism which is not seen in any other simulation, resulting in a final confirmation that is both tightly closed and protruded.

Further to this we conducted DCCM analysis to identify potential hot-spots of movement within the protein which may drive dissociation. Through plotting the absolute difference from the 30°C trajectory compared to the 37°C and 50°C trajectories it is possible to identify the amino acids with disrupted long distance correlations. Interestingly, PROA, B and E all showed significant changes in their long distance associations with PROA and PROB altering in correlation to each other whilst PROE also altered association with PROE (Figure 4). Further to this, plotting out the direction shows a loss of correlation between PROA and PROB, whilst the correlation of PROB to PROE shows a gain in correlation at 37°C. Plotting the top 20 amino acids with the most changed correlation values (Figure 4) identified a number of sites of interest, primarily at the interface of PROA and PROB, whilst some sites in PROC showed altered values potentially showing a hinge region forming, these are shown with the associated domain colouring (PROE correlation changes are coloured orange etc).
